# Editorial: Construction of smart materials for biomedical application

**DOI:** 10.3389/fbioe.2023.1278243

**Published:** 2023-08-23

**Authors:** Fei Chen, Jie Dong, Wen Sun, Daniele Di Iorio, Seraphine V. Wegner, Wenbin Zeng

**Affiliations:** ^1^ Xiangya School of Pharmaceutical Sciences, Central South University, Changsha, China; ^2^ State Key Laboratory of Fine Chemicals, Dalian University of Technology, Dalian, China; ^3^ Institute of Physiological Chemistry and Pathobiochemistry, University of Münster, Münster, Germany

**Keywords:** smart materials, stimuli-response, diagnosis and treatment, biomedical application, antibacterial

Smart materials, also known as responsive or intelligent materials, have opened up new frontiers in the biomedical field. Through extensive research and innovative engineering, these materials have been tailored to respond to specific triggers such as temperature, light, pH, pressure, and even biochemical signals (Lu et al., 2016). By intelligently responding to changes in their environment, smart materials can mimic natural biological processes, providing immense possibilities in medicine, drug delivery, tissue engineering, and diagnostics. The aim of this Research Topic was to broaden the knowledge and application in the areas of smart materials for biomedical applications, especially novel design and construction of bio-inspired, stimuli-responsive, multifunctional materials, or provide comprehensive insight into the advances of smart materials. In this pursuit of advancing smart materials for biomedical applications, interdisciplinary collaboration plays a vital role. The integration of diverse expertise from various scientific and medical fields can accelerate research progress and foster innovative solutions to the most pressing healthcare challenges.

Smart materials have the potential to revolutionize drug delivery systems, a transformative application within biomedical engineering. Traditional methods often struggle with precise targeting, leading to unwanted side effects or suboptimal therapeutic effects. Smart materials offer a solution by releasing therapeutic agents precisely at the desired site in response to specific triggers. In the realm of cancer research, nanotechnology, particularly nanomedicines, has gained prominence (Fan et al., 2017). Metal-based nanomedicines, responding to unique tumor conditions like acidity, hypoxia, and increased reactive oxygen species and glutathione levels, hold great potential for targeted drug delivery, modulating the tumor microenvironment, and combination therapies. Recent advancements focus on depleting intracellular glutathione to enhance therapeutic outcomes. These developments encompass smart metal nanomedicines, including inorganic nanomaterials, metal-organic frameworks (MOFs), and platinum-based nanomaterials, designed for glutathione (GSH)-responsive actions. Their application spans synergistic cancer therapies such as chemotherapy, photodynamic therapy (PDT), sonodynamic therapy (SDT), chemodynamic therapy (CDT), ferroptotic therapy, and radiotherapy. The review by Liu et al. explores these innovations, shedding light on mechanisms, sensitized therapy applications, and future prospects. This breakthrough holds promise for improved treatment efficacy, reduced dosages, fewer side effects, and enhanced quality of life for patients with chronic conditions. Furthermore, controlled and prolonged drug release could lead to fewer administrations, bolstering patient compliance and overall treatment success.

Smart materials drive diagnostic and imaging advancements by emitting detectable signals upon exposure to specific biomarkers, enabling highly sensitive tests for early disease detection and improved treatment monitoring. Monitoring blood glucose is critical, especially for conditions like diabetes, preventing severe complications. Traditional lab methods are impractical for home use, while glucometers are costly and inaccessible. A solution is a paper-based testing device using hydrogel and superabsorbent networks for enzyme-glucose reactions, offering a convenient diabetes diagnostic alternative (Cui et al., 2020). Hossain et al. introduced a nanocellulose-coated paper sensor immobilizing glucose oxidase enzyme for colorimetric glucose measurement. Elevated glucose levels induce pH changes, shifting color from red to orange. The sensor accurately measures glucose within the medical range (7–13 mM) across temperatures (4°C–40°C) with enzyme stability over a month. This study highlights nanocellulose sensors as cost-effective, robust, and sensitive diagnostics for various diseases.

Hand-arm vibration diseases are prevalents among workers using vibrating tools or materials, causing impaired circulation and nerve function (Fu et al., 2022). Factors such as vibration intensity and duration contribute to this condition. Prolonged exposure leads to microcirculatory and neural issues. Low-frequency, high-amplitude vibrations disrupt nerves and circulation, while high-frequency vibrations enhance tissue microcirculation. The utilization of wavelet analysis serves as a valuable tool for investigating physiological responses. The intricate interplay between inflammation, the NF-κB signaling pathway, and nitric oxide (NO) interaction assumes a significant role in shaping vascular health. Gaining a comprehensive understanding of the ramifications of vibrational stimuli becomes paramount in devising effective preventive strategies. Mu et al. investigated fingertip blood flow under different vibration amplitudes, using analysis to uncover vibration effects on blood circulation mechanisms. They assessed how local vibration intensity influences finger microcirculation. Through hand-transmitted vibration experiments and laser Doppler flowmetry, fingertip blood flow during vibration was measured. Low-frequency vibrations reduced blood flow, more pronounced in the vibrating hand than the contralateral hand. High amplitude vibrations triggered inflammatory responses in endothelial cells, altering their regulatory activity and affecting microcirculation. NF-κB expression increased with amplitude, highlighting the connection between vibration intensity and endothelial function.

Bacterial infections pose a significant global threat with escalating mortality rates and clinical complications. Antibiotic resistance and protective bacterial biofilms exacerbate the challenges, underscoring the need for innovative antibacterial agents. Conventional approaches are limited in addressing multi-drug-resistant infections, prompting the exploration of new antimicrobial strategies. Cationic polymers and nanomaterials, such as metal/metal oxide-based nanoparticles, have emerged as promising solutions due to their prolonged efficacy, stability, and reduced resistance (Gupta et al., 2019). These materials exhibit diverse antibacterial mechanisms, including loading antimicrobial agents, inducing physical or chemical damage, leveraging light-induced effects, generating reactive oxygen species (ROS), and synergizing multiple modes of action. Fan et al. explored recent progress in cationic polymers and nano-antibacterial materials, encompassing material choices, manufacturing methods, structural attributes, and performance efficacy, with particular attention to their underlying active components.

In conclusion, the construction of smart materials for biomedical applications represents a profound paradigm shift in healthcare. Through their dynamic and responsive properties, smart materials hold tremendous promise in drug delivery, tissue engineering, diagnostics, and medical imaging. The transformative potential of these materials can revolutionize patient care, improve treatment efficacy, and address some of the most significant medical challenges of our time.
